# Bacterial Community Expansion and Nutrient Activation Underlie Yield Improvement Following Diazotrophic Inoculant Application in Paddy Soil

**DOI:** 10.3390/microorganisms14071495

**Published:** 2026-07-08

**Authors:** Huai Shi, Guohong Liu

**Affiliations:** Institute of Resources, Environment and Soil Fertilizer, Fujian Academy of Agricultural Sciences, Fuzhou 350003, China

**Keywords:** diazotrophic inoculant, absolute quantification amplicon sequencing, soil microbial community, nutrient activation, inoculant legacy effect, rice yield

## Abstract

Reducing reliance on chemical nitrogen fertilizers while maintaining rice productivity is a key challenge in sustainable agriculture. In this study, a composite inoculant of three diazotrophic strains (*Paenibacillus azotifigens*, *Paenibacillus azotofixans*, and *Phytobacter diazotrophicus*) was applied by root drenching at the heading stage of field-grown rice. Soil physicochemical properties, rice yield, and soil bacterial and fungal communities were assessed at harvest using spike-in-based absolute quantification amplicon sequencing. Inoculation increased rice yield by 5.5% and significantly elevated soil nitrate nitrogen (NO_3_^−^-N) (+343%), with a trend toward higher available phosphorus, while total nitrogen, phosphorus, and carbon remained unchanged. Bacterial absolute abundance was approximately 2.6-fold higher in inoculated plots, while fungal abundance declined, resulting in a substantially elevated bacteria-to-fungi ratio; community composition and diversity indices showed no significant changes. The inoculant strains were not detectably enriched at harvest, yet functional groups associated with nitrification, nitrogen fixation, and organic matter decomposition were consistently elevated and positively associated with reactive nutrient fractions. These results suggest that diazotrophic inoculants may promote yield through transient microbial community activation and nutrient form transformation rather than persistent colonization, and highlight the value of absolute quantification for detecting inoculation-induced shifts in microbial community size.

## 1. Introduction

Rice (*Oryza sativa* L.) is one of the most important food crops in the world, providing the dietary staple for nearly three billion people. Sustaining high and stable yields is therefore critical to global food security [1]. Chemical nitrogen fertilizers have long been a cornerstone of high-yield rice production; however, the actual nitrogen use efficiency of rice typically ranges from only 30% to 50%, with substantial losses occurring through volatilization, leaching, and other pathways [2]. These losses contribute to soil acidification, increased greenhouse gas emissions, and eutrophication of water bodies [3,4]. Reducing dependence on chemical nitrogen fertilizers while maintaining rice productivity has thus become a central objective in sustainable agricultural research [5].

Microbial biofertilizers have received growing attention as a complement to chemical fertilizers [6]. Plant growth-promoting rhizobacteria (PGPR) can stimulate plant growth through multiple mechanisms, including biological nitrogen fixation, phosphate solubilization, phytohormone production, and suppression of plant pathogens [7,8]. Among these, diazotrophic bacteria convert atmospheric dinitrogen into plant-available inorganic nitrogen forms, representing an important pathway of biological nitrogen input in agricultural ecosystems [9,10]. Previous studies have demonstrated that inoculation of rice rhizospheres with diazotrophic bacteria can improve plant nitrogen nutrition, promote growth, and increase yield [11,12]. In open field environments, however, introduced strains typically face intense competition from indigenous microorganisms and environmental selection pressures, and their long-term stable colonization is often limited [13,14]. A growing body of evidence suggests that the growth-promoting effects of inoculants may not depend entirely on their persistent establishment, but may instead arise from transient ecological disturbances that reshape the composition and functional state of the indigenous microbial community, indirectly influencing soil nutrient cycling and crop performance [15,16]. This shift in understanding implies that characterizing the overall impact of inoculation on the native microbiome may be ecologically more informative than tracking the colonization dynamics of the introduced strains themselves.

The effects of diazotrophic inoculation on soil microbial community structure and function in paddy fields have been examined in previous studies [17,18,19], but most of this work has relied on relative abundance data from amplicon sequencing. Because relative abundances are subject to compositional data constraints, they do not accurately reflect changes in true microbial biomass and may therefore underestimate or misrepresent actual community responses to agricultural management [20,21]. Spike-in-based absolute quantification amplicon sequencing, by contrast, provides information on both community composition and absolute abundance [22], offering a new perspective for assessing changes in microbial carrying capacity and functional responses in agricultural soils [23]. Nevertheless, the application of this approach in field-based functional inoculant studies remains limited, particularly with respect to characterizing overall community expansion and associated functional shifts following inoculation.

In this study, a composite inoculant comprising three diazotrophic strains, *Paenibacillus azotifigens*, *Paenibacillus azotofixans*, and *Phytobacter diazotrophicus*, was applied by root drenching at the heading stage of rice. Soil physicochemical properties, rice yield, and bacterial and fungal community characteristics based on absolute quantification amplicon sequencing were determined at harvest. The specific objectives were to: (1) evaluate the effects of microbial inoculation on rice yield and soil nutrient status; (2) characterize the responses of soil microbial community absolute abundance and composition following inoculation; and (3) explore the potential relationships between microbial inoculation, soil nitrogen transformation, and microbial community restructuring.

## 2. Materials and Methods

### 2.1. Inoculant Preparation

The three strains used in this study, *Paenibacillus azotifigens*, *P*. *azotofixans*, and *Phytobacter diazotrophicus*, all possess biological nitrogen fixation capacity. Each strain was cultured separately in R2A liquid medium with shaking until the exponential growth phase, after which cell density was confirmed by plate counting. The three cultures were combined at a 1:1:1 (*v*/*v*/*v*) ratio to prepare the composite inoculant, which was then diluted with clean water to a final density of approximately 1.0 × 10^5^ CFU/mL prior to field application. At the heading stage, each plant received 10 mL of the inoculant suspension by root drenching, corresponding to 1.0 × 10^6^ CFU per plant.

### 2.2. Field Experimental Design

The field experiment was conducted in Fuzhou, Fujian Province, China, in 2024. The experimental soil was red earth-derived paddy soil (Waterlogogenic subgroup) with a clay loam texture. A randomized complete block design was used, with two treatments, diazotrophic inoculant (T) and water control (CK), each with three replicates. Each plot covered approximately 20 m^2^. Rice was managed according to local late-season cultivation practices. Compound fertilizer (N-P-K = 17-2.62-9.96, equivalent to N-P_2_O_5_-K_2_O = 17-6-12) was applied approximately one week after transplanting, with an equal second application approximately 20 days later. Fields were drained around 25 July and harvested around 25 August. At the heading stage, T plots received root drenching with the composite inoculant suspension, while CK plots received an equal volume of clean water.

### 2.3. Soil Sampling and Physicochemical Analysis

At harvest, soil samples were collected from the 0–20 cm layer in each plot using a five-point sampling strategy and thoroughly mixed to obtain a representative composite sample per plot. Subsamples were stored at −80 °C for microbial analysis, and air-dried subsamples were used for physicochemical measurements.

Soil pH was measured electrometrically in a 2.5:1 (*v*/*w*) water-to-soil suspension using a pH meter (PHS-3C, Leici, Shanghai, China). Total nitrogen (TN) and total carbon (TC) were determined using an elemental analyzer (Vario EL Cube, Elementar, Langenselbold, Germany). Total phosphorus (TP) was measured by ICP-MS (NexION 1000G, PerkinElmer, Shelton, CT, USA) following HNO_3_/HClO_4_ digestion. Available phosphorus (AP) was extracted by the Olsen method (0.5 M NaHCO_3_, pH 8.5) and measured by the same ICP-MS system. Ammonium nitrogen (NH_4_^+^-N) and nitrate nitrogen (NO_3_^−^-N) were extracted with 2 M KCl solution (1:5 soil-to-solution ratio) and determined by flow injection analysis (CleverChem Anna, DeChem-Tech. GmbH, Hamburg, Germany). Dissolved organic carbon (DOC) was measured with a TOC analyzer (TOC-L CPH, Shimadzu, Kyoto, Japan) after water extraction (1:5 soil-to-solution ratio). Soil iron (Fe) content was determined by ICP-MS following aqua regia digestion. All soil chemical determinations were performed in triplicate (*n* = 3).

### 2.4. Soil Microbial Community Analysis

Total soil DNA was extracted using the MP FastDNA SPIN Kit for Soil (MP Biomedicals, Solon, OH, USA) according to the manufacturer’s instructions. DNA quality was verified before submission to Shanghai Tianhao Biotechnology Co., Ltd. (Shanghai, Chian) for library preparation and sequencing. The V4–V5 region of the bacterial 16S rRNA gene was amplified using primers 515F (5′-GTGCCAGCMGCCGCGG-3′) and 907R (5′-CCGTCAATTCMTTTRAGTTT-3′), and the fungal ITS1 region was amplified using primers ITS1F (5′-CTTGGTCATTTAGAGGAAGTAA-3′) and ITS2R (5′-GCTGCGTTCTTCATCGATGC-3′) [24]. Sequencing was performed on the Illumina NovaSeq platform. Two biological replicates per treatment were used for amplicon sequencing (*n* = 2). Raw sequencing data were deposited in the NCBI Sequence Read Archive (accession number PRJNA1478032).

To obtain absolute abundance information, a known quantity of spike-in internal standard was added to each sample library prior to sequencing. The proportion of spike-in reads recovered in each sample was used to back-calculate the absolute copy numbers of bacteria and fungi, expressed as copies per gram of dry soil.

Raw sequencing data were quality-filtered and chimera-checked, and amplicon sequence variants (ASVs) were identified using the DADA2 (V1.36.0) pipeline before being merged into operational taxonomic units (OTUs) at 97% sequence similarity. Bacterial taxonomic annotation was performed against the SILVA database and fungal annotation against the UNITE database. Alpha diversity indices (Chao1 and Shannon index), beta diversity analysis based on Bray–Curtis dissimilarity (PCoA), and PERMANOVA tests were conducted in R (V4.4.1). Predicted ecological functions of the bacterial community were inferred using the FAPROTAX (V1.2.6) database based on OTU relative abundance data. Fungal functional guilds were predicted using FUNGuild (database version 1.2).

### 2.5. Statistical Analysis

Between-treatment differences were assessed using the Wilcoxon rank-sum test (*n* = 3), and effect sizes were calculated as Cohen’s *d*. Multivariate integration of soil physicochemical variables was performed by principal component analysis (PCA). Associations between microbial functional modules and environmental variables were assessed by Mantel tests, and pairwise correlations among soil parameters and between soil parameters and yield were evaluated by Pearson correlation analysis. All statistical analyses were performed in R (v4.5.3), with a significance threshold of *p* < 0.05.

## 3. Results

### 3.1. Inoculation Increased Rice Yield and Altered Soil Nutrient Availability

**Figure 1:**
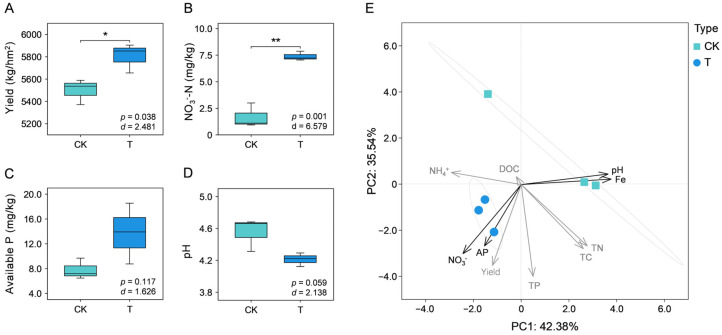
Effects of diazotrophic inoculant application on rice yield, selected soil physicochemical properties, and overall soil parameter differentiation. (**A**) Rice yield; (**B**) soil NO_3_^−^-N; (**C**) AP; (**D**) soil pH. Asterisks indicate statistical significance: * *p* < 0.05, ** *p* < 0.01 (Wilcoxon rank-sum test); Cohen’s *d* values are shown within each panel. (**E**) PCA of all measured soil physicochemical parameters. Bold arrows indicate variables with stronger loadings.

Rice yield was significantly higher in the inoculated treatment, increasing from 5497 ± 113.5 to 5802 ± 131.3 kg/hm^2^ (*p* = 0.038, Cohen’s *d* = 2.48), representing a 5.54% improvement (Table 1, Figure 1A).

Among the measured soil physicochemical parameters, the degree of treatment response varied considerably (Table 1, Figure 1B–E). Soil NO_3_^−^-N was significantly elevated in the inoculated treatment, increasing from 1.66 ± 1.15 mg/kg in CK to 7.37 ± 0.43 mg/kg in T (*p* = 0.001, Cohen’s *d* = 6.58), a 343% increase (Figure 1B). AP also showed an increasing trend, with a 76.9% increase and a large effect size (Cohen’s *d* = 1.63), though this did not reach statistical significance (*p* = 0.117), likely due to within-group variability and the limited sample size (Figure 1C). Soil pH declined in the inoculated treatment with a large effect size (Cohen’s *d* = 2.14) but did not reach significance (*p* = 0.059, Figure 1D). No significant differences were found for NH_4_^+^-N, TP, TN, TC, DOC, or Fe (*p* > 0.1, Table 1).

PCA integrating all soil physicochemical variables showed that PC1 and PC2 explained 42.38% and 35.54% of the total variance, respectively (Figure 1E). The two treatment groups showed a degree of separation in PCA space, with T samples distributed mainly along the negative PC1 axis and CK samples along the positive PC1 axis. NO_3_^−^-N, AP, pH, and Fe were identified as the primary contributors to sample differentiation, and yield was positively associated with NO_3_^−^-N and AP along the ordination axes.

### 3.2. Inoculation Expanded Bacterial Abundance Without Restructuring Community Composition

**Figure 2:**
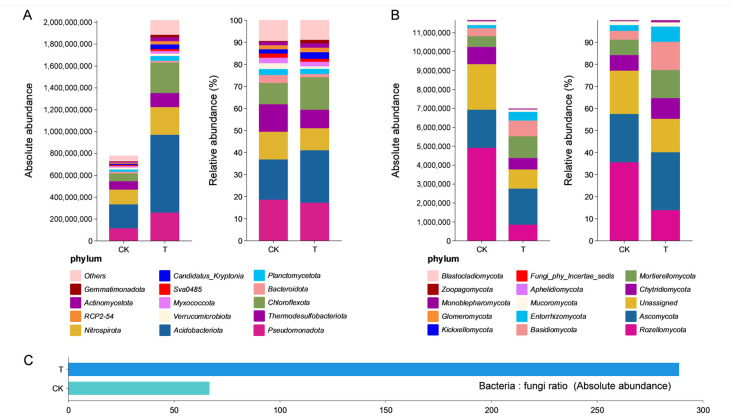
Absolute and relative abundance of soil bacterial and fungal communities and bacteria-to-fungi ratio in response to diazotrophic inoculant application. (**A**) Bacterial community composition shown as absolute abundance (copies/g dry soil, (**left**)) and relative abundance (%, (**right**)) at the phylum level. (**B**) Fungal community composition shown as absolute abundance (**left**) and relative abundance (**right**) at the phylum level. Each bar represents the mean of three replicates. (**C**) Bacteria-to-fungi ratio based on absolute abundance for CK and T treatments.

Absolute quantification showed that mean bacterial abundance in the inoculated treatment was 2018.6 ± 1114.1 × 10^6^ copies/g dry soil, approximately 2.6-fold higher than in CK (778.8 ± 178.2 × 10^6^ copies/g), although this difference did not reach statistical significance due to high within-group variability in T (*p* = 0.081, Figure 2A). Mean fungal abundance in T was 6.99 ± 2.90 × 10^6^ copies/g, lower than the 11.66 ± 8.09 × 10^6^ copies/g observed in CK (*p* = 0.400, Figure 2B). The bacteria-to-fungi ratio (B:F ratio) was approximately 289 in T, about 4.3-fold higher than in CK (B:F ≈ 67, Figure 2C), indicating that inoculation substantially altered the quantitative relationship between bacteria and fungi in the soil.

**Figure 3:**
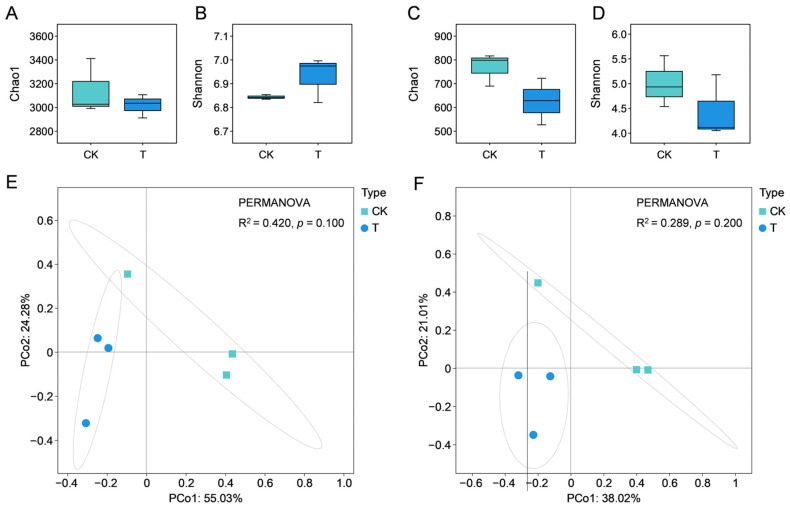
Alpha and beta diversity of soil bacterial and fungal communities in response to diazotrophic inoculant application. Bacterial alpha diversity: (**A**) Chao1 richness and (**B**) Shannon diversity index. Fungal alpha diversity: (**C**) Chao1 richness and (**D**) Shannon diversity index. Beta diversity based on Bray–Curtis dissimilarity: (**E**) PCoA of bacterial communities and (**F**) PCoA of fungal communities. PERMANOVA results are shown within each ordination panel. Ellipses represent 95% confidence intervals.

With respect to diversity, no significant differences in bacterial Shannon index or Chao1 were detected between treatments (Figure 3A,B), although the two indices changed in opposite directions: Chao1 was slightly lower in T than CK, while Shannon was slightly higher. Beta diversity analysis indicated that the treatment factor explained 42.0% of bacterial community variation (PERMANOVA, R^2^ = 0.420, *p* = 0.100, Figure 3E), with a trend toward separation between groups in PCoA space that did not reach significance. No significantly differentially abundant taxa were detected at the phylum level based on relative abundance (*p* > 0.05). However, absolute abundance profiles showed that copy numbers of all major phyla were elevated in T, suggesting a broad community-wide expansion rather than selective enrichment of particular lineages, a pattern that would be obscured by relative abundance alone (Figure 2A). No OTUs corresponding to the three inoculant strains were detected as significantly enriched at harvest, indicating that the introduced strains did not establish persistent detectable colonization by the time of sampling.

For fungi, both Chao1 and Shannon index were lower in T than CK (Figure 3C,D), with consistent directionality but no significant differences. Treatment explained 28.9% of fungal community variation (R2 = 0.289, *p* = 0.200, Figure 3F), and no significantly differentially abundant phyla were identified (*p* > 0.05, Figure 2B).

### 3.3. Nutrient Cycling-Related Functional Groups Were Consistently Enriched Following Inoculation

**Figure 4:**
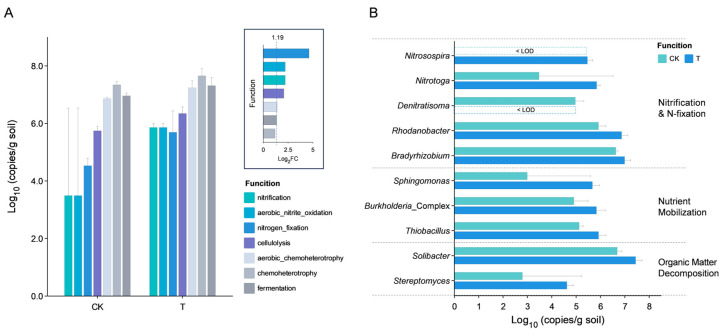
Predicted functional profiles and absolute abundances of key bacterial taxa in response to diazotrophic inoculant application. (**A**) Absolute abundance (log_10_-transformed, copies/g dry soil) of FAPROTAX-predicted functional categories in CK and T. The inset shows the Log_2_FC of each functional category in T relative to CK; the dashed line indicates the Log_2_FC of chemoheterotrophy as a baseline reference for overall heterotrophic metabolism. (**B**) Absolute abundances (log_10_-transformed) of selected bacterial genera grouped by functional module: Nitrification & N-fixation, Nutrient Mobilization, and Organic Matter Decomposition. Error bars represent standard deviation (*n* = 3). “<LOD” indicates that abundance was below the limit of detection in that treatment.

FAPROTAX-based functional prediction showed that all selected functional pathways were elevated in the inoculated treatment, although differences did not reach statistical significance (Wilcoxon test, *p* = 0.081, Figure 4A). Using chemoheterotrophy as a baseline for overall heterotrophic metabolism (Log_2_FC = 1.19), nitrogen transformation-related functions showed more pronounced increases. Nitrogen fixation showed the largest fold change (Log_2_FC = 4.62, FC = 24.6), and nitrification/aerobic nitrite oxidation also increased substantially (Log_2_FC = 2.22), while nitrate respiration and nitrate reduction pathways changed to a lesser extent. Carbon cycling functions were also elevated: cellulolysis increased 4.23-fold (Log_2_FC = 2.08), fermentation 2.57-fold (Log_2_FC = 1.36), and aerobic chemoheterotrophy 2.64-fold (Log_2_FC = 1.40).

Consistent with these functional trends, the absolute abundances of key bacterial taxa associated with these functions were also higher in the inoculated treatment (Figure 4B). The ammonia oxidizer *Nitrosospira* was below the detection limit in CK but detectable in T; the nitrite oxidizer Candidatus *Nitrotoga* increased 4.7-fold (Log_2_FC = 2.22); and the nitrogen-fixing taxon *Bradyrhizobium* also showed higher abundance (Log_2_FC = 1.34). Among denitrification-related taxa, *Denitratisoma* was only detectable in CK, while *Rhodanobacter* was more abundant in T (Log_2_FC = 1.52), suggesting a complex response of the denitrification community to inoculation. Taxa associated with nutrient mobilization and organic matter turnover, including the *Burkholderia–Caballeronia–Paraburkholderia* complex, *Solibacter*, *Sphingomonas*, *Streptomyces*, and *Thiobacillus*, all showed higher abundances in the inoculated treatment.

These functional and taxonomic changes were characterized by consistent directional trends rather than statistically significant differences. Fungal functional composition predicted by FUNGuild is summarized in Table S2.

### 3.4. Microbial Functional Groups Were Associated with Active Soil Nutrient Pools

Pearson correlation analysis showed that rice yield was positively associated with both NO_3_^−^-N (r = 0.796, *p* = 0.058) and TP (r = 0.800, *p* = 0.056), and NO_3_^−^-N and AP were also correlated with each other (r = 0.719, *p* = 0.107).

**Figure 5:**
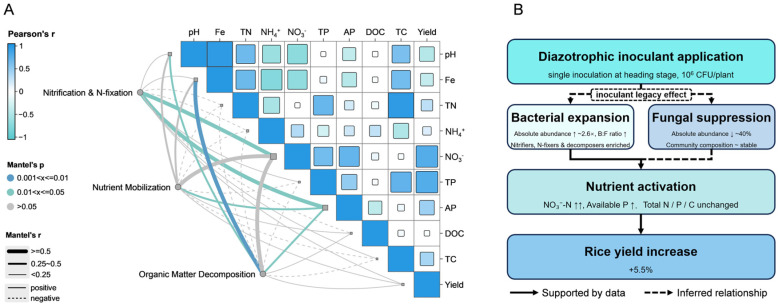
Associations among microbial functional modules, soil physicochemical properties, and rice yield, and a proposed mechanistic framework. (**A**) Mantel test results linking three microbial functional modules with soil physicochemical variables (line width indicates Mantel’s r; line color indicates Mantel’s *p*), overlaid on a Pearson correlation matrix of soil parameters and rice yield (color intensity and square size reflect correlation strength). (**B**) Conceptual diagram of the proposed pathway linking diazotrophic inoculant application to rice yield improvement. Solid arrows indicate relationships supported by consistent trends or statistically significant results in this study; dashed arrows indicate inferred relationships.

The three functional modules showed distinct patterns of association with environmental variables (Figure 5A). The Nitrification & N-fixation module was significantly and positively correlated with both NO_3_^−^-N (r = 0.671, *p* = 0.049) and AP (r = 0.751, *p* = 0.013). The Nutrient Mobilization module was significantly correlated with AP (r = 0.493, *p* = 0.039) and showed a strong trend of association with NO_3_^−^-N (r = 0.663, *p* = 0.069). The Organic Matter Decomposition module was significantly correlated with AP (r = 0.457, *p* = 0.046), pH (r = 0.492, *p* = 0.021), and Fe (r = 0.521, *p* = 0.007), and also showed a positive trend with NO_3_^−^-N (r = 0.691, *p* = 0.056). All three modules showed positive but non-significant associations with yield (r = 0.157–0.207, *p* > 0.05).

Overall, all three functional modules were more strongly associated with reactive nutrient indicators such as NO_3_^−^-N and AP than with total nutrient pools such as TN, TP, and TC, consistent with the nutrient response patterns observed under inoculation. A conceptual diagram integrating these findings into a proposed mechanistic framework is presented in Figure 5B.

## 4. Discussion

### 4.1. Inoculation Shifted Soil Nutrient Speciation and Was Associated with Yield Improvement

Under field conditions, application of the composite diazotrophic inoculant was associated with a 5.54% increase in rice yield, which falls within the range reported in the literature. Previous studies have shown that diazotroph-based biofertilizers produce an average yield increase of approximately 14.5% [25], while composite diazotrophic inoculants applied to rice under field conditions typically yield increases of 5–30%, with considerable variation depending on strain combination, inoculation method, and field conditions [26,27,28]. Among the three strains used in this study, *Phytobacter diazotrophicus* has been reported to possess multiple plant growth-promoting properties including nitrogen fixation and phosphate solubilization [29]. Species-level studies on *Paenibacillus azotifigens* and *P. azotofixans* are currently limited, but the genus *Paenibacillus* is a well-recognized PGPR group, and nitrogen fixation, phosphate solubilization, and phytohormone production have been documented across multiple species within the genus [30,31], suggesting that these strains have the potential to promote plant growth through several complementary mechanisms.

With respect to soil nutrients, TN, TP, and TC did not differ significantly between treatments, indicating that inoculation did not substantially alter the overall size of the soil nutrient pool. The pronounced increase in NO_3_^−^-N and the trend toward higher AP, however, indicate that inoculation was accompanied by a shift in nutrient speciation and bioavailability, with elevated reactive nutrient fractions against a background of largely unchanged total nutrient stocks. This pattern is consistent with observations from other PGPR studies, where microbial inoculation primarily promotes nutrient form transformation and turnover rather than net nutrient addition [32].

Among all measured parameters, NO_3_^−^-N showed the strongest response to inoculation and was positively associated with yield, suggesting that improved nitrogen availability may be an important contributor to the observed yield increase. Following drainage at the grain-filling stage, the shift from flooded anaerobic to aerobic soil conditions promotes nitrification and favors NO_4_^+^-N oxidation and NO_3_^−^-N accumulation [33]. At this growth stage, plant demand for additional nitrogen uptake is low, as grain filling relies primarily on remobilization of nitrogen accumulated in vegetative tissues [34], which further allows nitrification-derived NO_3_^−^-N to accumulate in the soil. Because both treatments followed the same water management and fertilization schedule, the substantially higher NO_3_^−^-N in the inoculated plots more likely reflects a treatment-associated difference in soil biological activity rather than differences in drainage timing or crop nitrogen uptake.

Soil pH showed a declining trend in the inoculated treatment, which is directionally consistent with the acidifying effect of nitrification. In acidic soils, lower pH generally promotes adsorption of phosphorus onto iron and aluminum oxides, which would be expected to reduce phosphorus availability [35]. The observed increase in AP therefore more likely reflects direct microbial phosphate solubilization rather than an indirect chemical effect mediated by pH change.

### 4.2. Inoculation Expanded the Bacterial Community Without Persistent Inoculant Colonization

Absolute quantification revealed that soil bacterial abundance was approximately 2.6-fold higher in the inoculated treatment than in CK, while fungal abundance declined to approximately 60% of the control level, suggesting that inoculation had a measurable effect on the overall size of the microbial community. In contrast, alpha and beta diversity indices for both bacteria and fungi showed no significant differences between treatments, and no significantly differentially abundant taxa were detected at the phylum or genus level. Absolute abundance profiles indicated that copy numbers across all major bacterial phyla were elevated in the inoculated treatment, with no statistically significant selective enrichment at the phylum level. Within the scope of the present data, inoculation was therefore associated more with an overall expansion in microbial community size than with directional changes in community composition. This finding also highlights the value of absolute quantification approaches that, when total microbial biomass changes substantially, relative abundance data alone cannot capture shifts in community size [36,37].

The decline in fungal absolute abundance may reflect increased competition from an expanded bacterial community, inhibitory effects of metabolites produced by the inoculant strains or proliferating bacteria, or differences in microenvironmental conditions among plots.

No OTUs corresponding to the three inoculant strains were detected as significantly enriched at harvest. Similar outcomes have been reported in other field inoculation studies. Introduced strains typically decline in abundance over time due to competition from indigenous microorganisms, resource limitation, and environmental filtering [16]. It has been proposed that even transient inoculant activity can restructure native microbial communities by altering competitive dynamics or releasing ecological niches, activating the proliferation of indigenous functional taxa and driving community-level changes that persist after the inoculant itself has declined, a phenomenon referred to as the inoculant legacy effect [38]. Sampling in this study was conducted approximately 30 days after inoculation, at which point the inoculant strains were no longer detectable, yet bacterial community size and soil nutrient status both showed consistent changes associated with the inoculation treatment, which is in line with this hypothesis. The possibility that inoculant strains persisted at low abundance in microsites not captured by the sampling scheme, such as deeper soil layers, specific root zones, or soil aggregates, cannot be entirely excluded, but the overall pattern suggests that sustained dominance is not a prerequisite for downstream ecological effects.

The opposing trends in bacterial and fungal abundance resulted in a substantial increase in the B:F ratio, from approximately 67 in CK to approximately 289 in T. Bacterially dominated communities are generally associated with faster nutrient turnover, while fungal-dominated communities are more commonly linked to slower organic matter accumulation [39]. The elevated B:F ratio is therefore directionally consistent with the inference that bacterially mediated nutrient cycling processes were enhanced in the inoculated treatment.

### 4.3. Inoculation Enriched Bacterial Nutrient Cycling Functions Without Altering Fungal Functional Composition

Consistent with the overall expansion of the bacterial community, multiple nutrient cycling-related predicted functions and key bacterial taxa showed uniform increases in the inoculated treatment. Nitrogen transformation showed the most prominent changes: FAPROTAX prediction indicated increases in both nitrogen fixation and nitrification/aerobic nitrite oxidation functions, while nitrate reduction and nitrate respiration pathways changed to a lesser degree. At the taxon level, the co-expansion of the ammonia oxidizer *Nitrosospira* and the nitrite oxidizer *Nitrotoga* is taxonomically consistent with enhanced complete nitrification, providing indirect community-level support for the observed accumulation of NO_3_^−^-N in the inoculated soil.

The nitrogen-fixing genus *Bradyrhizobium* and the predicted nitrogen fixation function both increased consistently, yet soil TN did not change detectably. Because FAPROTAX reflects functional potential inferred from taxonomic annotation rather than actual process rates, these changes are more appropriately interpreted as an increase in the abundance of nitrogen-fixing microorganisms rather than direct evidence of elevated biological nitrogen fixation rates. Furthermore, in agricultural soils with relatively adequate nitrogen supply, diazotrophic bacteria tend to downregulate nitrogen fixation activity and instead promote plant growth through phosphate solubilization, siderophore production, and phytohormone synthesis [40]. The multifunctional growth-promoting potential of taxa such as *Bradyrhizobium* is well supported in the literature [41,42].

Beyond nitrogen cycling, carbon turnover-related functions including cellulolysis, chemoheterotrophy, and fermentation were also elevated, along with higher abundances of the *Burkholderia–Caballeronia–Paraburkholderia* complex, *Sphingomonas*, *Solibacter*, and *Streptomyces*, which are broadly involved in organic matter decomposition, nutrient release, and rhizosphere metabolism [43,44,45,46].

FAPROTAX is valuable for identifying functional differences between treatments and revealing functional trends, but its coverage of phosphate solubilization and organic phosphorus mineralization is relatively limited [47], so phosphorus transformation processes may not be fully captured in the functional predictions. The measured increase in AP nevertheless provides independent soil chemical evidence that phosphorus activation and nitrogen transformation may have occurred in concert, together forming the functional basis of the nutrient activation effect associated with inoculation.

In contrast to the bacterial community, FUNGuild-based prediction showed no consistent broad-scale restructuring of fungal functional composition, and a large proportion of sequences remained functionally unassigned (Table S2). Nevertheless, fungal guilds containing plant pathogenic functions, including Plant Pathogen, Plant Pathogen-Undefined Saprotroph, and Plant Pathogen-Wood Saprotroph, were consistently lower in the inoculated treatment, a pattern also reflected in the decline of Pathotroph and Pathotroph-Saprotroph trophic modes. Suppression of plant pathogenic fungi by PGPR inoculants has been reported previously and may represent an additional contributor to the observed yield improvement [48]. Given the overall decline in fungal abundance and the high proportion of unassigned sequences, deeper interpretation of fungal functional responses remains limited.

### 4.4. Microbial Functional Shifts Co-Occurred with Enhanced Active Nutrient Availability and Yield

Mantel tests showed that all three microbial functional modules, Nitrification & N-fixation, Nutrient Mobilization, and Organic Matter Decomposition, were more strongly associated with reactive nutrient indicators such as NO_3_^−^-N and AP than with total nutrient pools including TN, TP, and TC. This pattern is consistent with the nutrient response observed in Section 3.1, where inoculation was primarily associated with changes in reactive nutrient fractions rather than total nutrient accumulation.

The Nitrification & N-fixation module showed strong positive associations with both NO_3_^−^ and AP. Coupling between nitrogen and phosphorus cycling at the microbial functional level has been reported previously, and experimental evidence supports the capacity of taxa such as *Bradyrhizobium* and *Burkholderia* to simultaneously perform nitrogen transformation and phosphate solubilization [49,50]. This functional coupling is consistent with the concurrent increases in NO_3_^−^ and AP observed in the inoculated treatment and may account for the strong correlation between the nitrogen transformation module and AP. The Organic Matter Decomposition module was associated with Fe, pH, and reactive nutrient indicators, reflecting the close coupling between organic matter turnover and soil redox conditions. In paddy soil ecosystems, iron cycling, organic matter transformation, and nutrient release are interconnected processes that collectively influence the bioavailability of nitrogen and phosphorus [51].

With respect to yield-associated factors, the positive association between yield and NO_3_^−^-N has a clear physiological basis, as NO_3_^−^-N is a directly plant-available form of inorganic nitrogen and its increase directly reflects improved nitrogen availability [52]. Total phosphorus also showed a similarly strong positive association with yield, but TP reflects long-term phosphorus supply potential rather than immediate plant nutrient availability, and its agronomic effect is generally expressed through reactive fractions such as AP [53]. The relatively high within-group variability in AP in this study may have reduced the statistical strength of its association with yield. Overall, the most pronounced changes following inoculation were in reactive nutrient fractions, particularly NO_3_^−^-N and AP, rather than in total nutrient pools, and the core factor most consistently associated with the yield increase remains the improvement in nitrogen availability, likely accompanied by enhanced phosphorus mobilization.

Taken together, the results reveal a directionally consistent pattern across bacterial community expansion, enrichment of nutrient cycling-related functional groups, increases in reactive nutrient fractions, and yield improvement in the inoculated treatment. This pattern supports the hypothesis that microbially mediated nutrient activation is an important pathway through which diazotrophic inoculants exert their agronomic effects. A proposed mechanistic framework is summarized in Figure 5B. This mechanism warrants further investigation through time-series sampling and functional gene-level verification.

## 5. Conclusions

Inoculation with a composite diazotrophic inoculant increased rice yield by approximately 5.5% under field conditions. Absolute quantification amplicon sequencing showed that inoculation altered overall microbial community size rather than its compositional structure. The inoculant strains were not detectably enriched at harvest, yet nutrient cycling-related functional groups, particularly those associated with nitrification and nutrient mobilization, were consistently elevated and strongly associated with increased reactive nitrogen and phosphorus fractions. These findings support a scenario in which transient inoculant colonization triggers lasting microbial community restructuring and nutrient activation, ultimately promoting rice yield. The use of absolute quantification was critical to revealing community-scale changes that relative abundance analysis alone would have missed. Future work with larger sample sizes, time-series sampling, and direct functional gene quantification is needed to establish the mechanistic basis of these effects more rigorously.

## Figures and Tables

**Table 1:** Soil physicochemical properties and rice yield in response to diazotrophic inoculant application.

Parameter	CK	T	*p*-Value	Change (%)	Cohen’s *d*
pH	4.55 ± 0.21	4.21 ± 0.09	0.059	−7.47	**2.14**
Fe (g/kg)	39.44 ± 8.93	27.41 ± 1.30	0.142	−30.49	**1.89**
Total P (g/kg)	0.59 ± 0.14	0.70 ± 0.02	0.281	+19.37	**1.18**
NH_4_^+^-N (mg/kg)	2.18 ± 0.51	2.50 ± 0.34	0.426	+14.49	0.72
NO_3_^−^-N (mg/kg)	1.66 ± 1.15	7.37 ± 0.43	**0.001**	+343.07	**6.58**
Available P (mg/kg)	7.75 ± 1.69	13.71 ± 4.90	0.117	+76.90	**1.63**
DOC (mg/kg)	171.15 ± 20.24	169.92 ± 8.81	0.928	−0.72	0.08
TN (g/kg)	2.48 ± 0.64	2.40 ± 0.26	0.848	−3.26	0.17
TC (g/kg)	26.59 ± 5.87	26.26 ± 2.79	0.663	−1.25	0.18
Yield (kg/hm^2^)	5497.5 ± 113.5	5802 ± 131.3	**0.038**	+5.54	**2.48**

Values are presented as mean ± standard deviation (*n* = 3). Statistical significance was assessed using the Wilcoxon rank-sum test; bold *p*-values indicate statistical significance (*p* < 0.05). Cohen’s *d* was calculated as a measure of effect size; bold values indicate large effect sizes (*d* > 0.8). CK: water control; T: diazotrophic inoculant treatment.

## Data Availability

The original data presented in the study are openly available in the NCBI Sequence Read Archive at PRJNA1478032.

## Supplementary Materials

The following supporting information can be downloaded at: https://www.mdpi.com/article/10.3390/microorganisms14071495/s1, Table S1: OTU search results for the inoculated bacteria. Table S2: Fungal functional guilds predicted by the FUNGuild database.
