# Supplementary material for: Bacterial Community Expansion and Nutrient Activation Underlie Yield Improvement Following Diazotrophic Inoculant Application in Paddy Soil
Source: Microorganisms. 2026 Jul 8;14(7):1495. doi: 10.3390/microorganisms14071495 (PMC13413859; doi:10.3390/microorganisms14071495)
Supplement: Supplementary file 1 [file microorganisms-14-01495-s001.zip › microorganisms-4408880-supplementary.pdf]

Table S1. OTU search results for the inoculated bacteria.

#OTU ID	CK-1	CK-2	CK-3	T-1	T-2	T-3	OUT size	Taxonomy
ASV4785	0	0	13674	0	0	0	13674	Bacteria{superkingdom}(100);Bacillota{phylum}(100);Bacilli{class}(100);Paenibacillales{order}(100);Paenibacillaceae{family}(100);Paenibacillus{genus}(100);Unassigned{species}(0)
ASV6673	10081	0	0	0	0	0	10081	Bacteria{superkingdom}(100);Bacillota{phylum}(100);Bacilli{class}(100);Paenibacillales{order}(100);Paenibacillaceae{family}(100);Paenibacillus{genus}(100);Unassigned{species}(0)
ASV8294	5770	0	0	0	0	0	5770	Bacteria{superkingdom}(87);Bacillota{phylum}(87);Bacilli{class}(87);Paenibacillales{order}(87);Paenibacillaceae{family}(87);Paenibacillus{genus}(87);Unassigned{species}(0)
ASV8770	0	0	0	0	0	18530	18530	Bacteria{superkingdom}(100);Bacillota{phylum}(100);Bacilli{class}(100);Paenibacillales{order}(100);Paenibacillaceae{family}(100);Paenibacillus{genus}(100);Unassigned{species}(0)
ASV9349	0	0	0	0	0	14319	14319	Bacteria{superkingdom}(100);Bacillota{phylum}(100);Bacilli{class}(100);Paenibacillales{order}(100);Paenibacillaceae{family}(100);Paenibacillus{genus}(100);Unassigned{species}(0)
ASV9472	3366	0	0	0	0	0	3366	Bacteria{superkingdom}(93);Bacillota{phylum}(93);Bacilli{class}(93);Paenibacillales{order}(93);Paenibacillaceae{family}(93);Paenibacillus{genus}(93);Unassigned{species}(0)
ASV9706	0	0	0	4398	0	0	4398	Bacteria{superkingdom}(100);Bacillota{phylum}(100);Bacilli{class}(100);Paenibacillales{order}(100);Paenibacillaceae{family}(100);Paenibacillus{genus}(100);Unassigned{species}(0)
ASV10090	0	1638	0	0	0	0	1638	Bacteria{superkingdom}(100);Bacillota{phylum}(100);Bacilli{class}(100);Paenibacillales{order}(100);Paenibacillaceae{family}(100);Paenibacillus{genus}(100);Unassigned{species}(0)

Table S2. Fungal functional guilds predicted by FUNGuild database

Trophic_Mode	CK-1	CK-2	CK-3	T-1	T-2	T-3
Pathotroph	103093	13936	166694	11514	13760	30864
Pathotroph-Saprotroph	395191	25728	697454	1919	30963	13228
Pathotroph-Saprotroph-Symbiotroph	33138	10720	47042	15350	12042	26456
Saprotroph	517925	229406	1371385	424016	419735	573205
Saprotroph-Symbiotroph	112912	135070	387587	97850	61928	2486842
Symbiotroph	49093	9648	57269	5757	18921	17637
Undefined	16172206	1987474	12469258	3706817	6118796	6891690
**Guild**	**CK-1**	**CK-2**	**CK-3**	**T-1**	**T-2**	**T-3**
Algal Parasite-Bryophyte Parasite-Fungal Parasite-Undefined Saprotroph	240551	8576	379405	0	3440	0
Animal Pathogen	1227	0	20454	3838	1720	4409
Animal Pathogen-Endophyte-Endosymbiont-Epiphyte-Soil	2455	1072	0	5756	0	0

Saprotroph-Undefined Saprotroph						
Animal Pathogen-Endophyte-Epiphyte-Fungal Parasite-Plant Pathogen-Wood Saprotroph	23319	6432	30680	7675	12042	26456
Animal Pathogen-Plant Pathogen-Undefined Saprotroph	0	0	25567	0	0	0
Animal Pathogen-Undefined Saprotroph	0	1072	10227	0	0	0
Arbuscular Mycorrhizal	2455	6432	14317	1919	10320	0
Dung Saprotroph	0	0	0	1919	0	0
Dung Saprotroph-Nematophagous	0	3216	0	3837	10321	0
Dung Saprotroph-Plant Saprotroph	46637	20368	57268	21105	51607	83776
Dung Saprotroph-Soil Saprotroph-Wood Saprotroph	8591	0	18408	0	1720	0
Dung Saprotroph-Undefined Saprotroph	6137	26800	13295	47966	58489	105823
Dung Saprotroph-Undefined Saprotroph-Wood Saprotroph	25774	1072	29657	138140	24083	4409
Ectomycorrhizal	4909	1072	7159	3838	3440	17637
Ectomycorrhizal-Fungal Parasite-Plant Pathogen-Wood Saprotroph	0	0	1023	0	0	0
Ectomycorrhizal-Undefined Saprotroph	3682	1072	20453	1919	3440	0
Ectomycorrhizal-Wood Saprotroph	0	0	7159	0	0	0
Endophyte	3682	2144	0	0	0	0
Endophyte-Lichen Parasite-Plant Pathogen-Undefined Saprotroph	7364	3216	16362	1919	0	0
Endophyte-Litter Saprotroph-Soil Saprotroph-Undefined Saprotroph	109230	133998	358952	95931	56768	2486842
Endophyte-Litter Saprotroph-Wood Saprotroph	0	0	0	0	1720	0
Epiphyte	2455	0	19430	0	5161	0
Fungal Parasite	0	0	2045	1919	0	0
Fungal Parasite-Litter Saprotroph-Undefined Saprotroph	2455	0	0	0	0	0
Fungal Parasite-Plant Pathogen-Plant Saprotroph	66274	2144	13295	0	1720	0
Litter Saprotroph-Soil Saprotroph-Wood Saprotroph	0	0	3068	0	0	0
Orchid Mycorrhizal	35592	0	16363	0	0	0
Plant Pathogen	101866	13936	148286	5757	12040	26455
Plant Pathogen-Undefined Saprotroph	8591	3216	165671	0	0	0
Plant Pathogen-Wood Saprotroph	77320	10720	99198	1919	25803	13228

Plant Saprotroph	2455	0	0	0	0	0
Plant Saprotroph-Wood Saprotroph	12274	2144	32725	40292	24083	127869
Soil Saprotroph-Undefined Saprotroph	9818	4288	56246	0	6881	0
Wood Saprotroph	69956	33231	81814	36453	30964	26456
Undefined Saprotroph	336283	138287	1078904	134304	211587	224872
Undefined	16172206	1987474	12469258	3706817	6118796	6891690
